# An infectious clone of enterovirus 71(EV71) that is capable of infecting neonatal immune competent mice without adaptive mutations

**DOI:** 10.1080/22221751.2020.1729665

**Published:** 2020-02-21

**Authors:** Huiying Zhang, Zhigang Song, Jingyi Zou, Yanling Feng, Jing Zhang, Lehao Ren, Xiaonan Zhang, Yunwen Hu, Zhenghong Yuan, Zhigang Yi

**Affiliations:** a Department of Pathogen Diagnosis and Biosafety, Shanghai Public Health Clinical Center, Fudan University, Shanghai, People’s Republic of China; b Key Laboratory of Medical Molecular Virology (MOE/NHC/CAMS), School of Basic Medicine, Shanghai Medical College, Fudan University, Shanghai, People’s Republic of China; c Department of Clinical Pathology, Shanghai Public Health Clinical Center, Fudan University, Shanghai, People’s Republic of China

**Keywords:** Enterovirus 71, infectious clone, mouse model, adaptive mutation, myolysis, pulmonary pathology

## Abstract

Enterovirus 71 (EV71) is a major pathogen that causes hand, foot and mouth disease (HFMD), which is a life threatening disease in certain children. The pathogenesis of EV71-caused HFMD is poorly defined due to the lack of simple and robust animal models with severe phenotypes that recapitulate symptoms observed in humans. Here, we generated the infectious clone of a clinical isolate from a severe HFMD patient. Virus rescued from the cDNA clone was infectious in cell lines. When administrated intraperitoneally to neonatal ICR, BALB/c and C57 immune competent mice at a dosage of1.4 × 10^4^ pfu per mouse, the virus caused weight loss, paralysis and death in the infected mice after 4–5 days of infection. In the infected mice, detectable viral replication was detected in various tissues such as heart, liver, brain, lung, kidney, small intestine, leg skeletal muscle and medulla oblongata. The histology of the infected mice included massive myolysis, glomerular atrophy, villous blunting in small intestine, widened alveolar septum, diminished alveolar spaces and lymphocytes infiltration into the lung. By using the UV-inactivated virus as a control, we elucidated that the virus first amplified in the leg skeletal muscle tissue and the muscle tissue served as a primary viral replication site. In summary, we generated a stable EV71 infectious clone that is capable of infecting neonatal immune competent mice without adaptive mutations and provide a simple, valuable animal model for the studies of EV71pathogenesis and therapy.

## Introduction

Enterovirus 71 (EV71) causes hand, foot and mouth disease (HFMD) that is associated with severe neurological complications and even death in infants and young children all over the world [1]. EV71 is a family member of *Picornaviridae*. Its 7.4 kb positive-sense genome encodes a single open reading frame, which is translated into a polypeptide and cleaved into at least 11 individual proteins in the following order: 5′-VP4-VP2-VP3-VP1-2A-2B-2C-3A-3B-3C-3D-3′. The VP1, VP2, VP3 and VP4 are structural proteins [2, 3]. VP1 protein plays an important role in binding to cellular receptors [4]. Its GH cervical ring structure is the site for interaction with SCARB2 receptor [5].

The pathogenesis of EV71 caused severe diseases is poorly defined due to the lack of simple, stable animal models. The current EV71 animal models cannot fully recapitulate similar disease patterns and symptoms observed in humans [6]. Simple and stable small animal models are also required for developing anti-viral drugs. There are several EV71 animal models including non-human primate such as cynomolgus, rhesus and green monkey [7–9]. Reported EV71 mouse models include innate immunity deficient mice such as A129, AG129 mice [10, 11] and transgenic mouse that expresses human SCARB2 or PSGL-1 receptors [12, 13]. However, the limited availability of these models may hamper their broader applications. Meanwhile, to establish EV71 mouse models, EV71 needs to be adapted as a mouse-adapted strain [12, 14–17] to get infectivity in the animals. Alternatively, a high dose of virus (as high as 10^6^–10^7^ plaque-forming unit (PFU) per mouse) without adaptive mutations is needed to infect neonatal mice, transgenic mice or immunodeficient mice [11, 18–20].

In this study, we generated infectious clone of an EV71 clinical isolate. Virus rescued from the cDNA clone was infectious in cell lines, and when administrated intraperitoneally to immune competent neonatal ICR, BALB/c and C57 mice at a dosage of 1.4 × 10^4^ pfu per mouse, caused weight loss, paralysis and death in the infected mice after 4–5 days of infection. This simple, stable and efficient mouse model may greatly benefit pathogenesis studies of enterovirus-associated diseases and anti-enterovirus drug development.

## Material and methods

### Ethical

Use of mice was reviewed and approved by the Ethics committee of the Shanghai Public Health Clinical Center.

### Cell lines, virus and mice

Human muscular rhabdomyosarcoma (RD) cells and African Green Monkey Kidney (Vero) cells were maintained in Dulbecco’s modified eagle medium (DMEM) containing 10% FBS supplemented with 1% penicillin, and streptomycin (Hyclone, USA).

Enterovirus 71 strain SHAPHC695F/SH/CHN/10 (695F)(GenBank: JQ736684.2) was isolated from faecal sample of a 1.8-year-old patient in Shanghai public health clinical centre (SHPHC) in 2010 in RD cells [21]. Briefly, 400 μl of clarified faecal specimens were filtered with 0.22 μm filter and then added into cells on 6-well plate. When the cells appeared obvious cytopathic effect (CPE), the supernatant were collected and recorded as EV71 695F C+1. For virus expansion, 200 μl supernatant was used to infect naïve RD cells, and the supernatant from the first passage was recoded as EV71 695F C+2 (Parental EV71 695F).

Specific-pathogen-free ICR, C57 and Balb/C mice were maintained in the animal facility of Shanghai Public Health Clinical Center SHPHC. The animals were cared in accordance with the guidelines of the animal centre of SHPHC.

### Plasmids

To generate the infectious cDNA clones, Vero cells were infected with EV71 695F C+1. After 24 h, cells were lysed and total RNAs were extracted using Qiagen RNeasy Mini Kit (Qiagen). RNAs were reversely transcribed with random primer by using superscript II reverse transcription kit (Invitrogen) according to the manufactures’ guide. Four fragments covering the whole genome of EV71 with overlapping restriction enzyme sites were amplified, and cloned into pANCR vector stepwise by restriction enzyme digestion. A T7 promoter was flanked to the 5′ of the viral genome and a ployA (A30) was added to the 3′ of the viral genome. A Hind III site was added behind the polyA sequence. The assembled plasmid was named as pEV71-js1. To generate Nanoluc (Nluc) reporter infectious clones, the sequence encoding the Nluc was inserted in frame with viral open reading frame (ORF) and upstream the VP4 by fusing PCR. A sequence encoding the 2A protease recognition site (AITTL) was duplicated and inserted downstream of Nluc to facilitate the cleavage of Nluc from the viral polyprotein. The VP1 E145G and 3C C147 mutations were introduced into infectious clones by fusing PCR mediated mutagenesis. All the plasmid sequences were proofed by Sanger sequencing. The details of the primers used for plasmid construction are available upon request.

### In-vitro transcription

For *in-vitro* transcription, virus plasmids were linearized with HindIII and full-length RNA transcripts were synthesized using a MEGAscript T7 transcription kit (Thermo Fisher Scientific, Waltham, MA) following the manufacturer’s instructions. The RNAs were purified by RNeasy Mini kit (Qiagen) and dissolved in RNase-free H_2_O and quantified by determining the A260 absorbance.

### Viral RNA transfection

Prior to transfection, 0.75 × 10^5^ cells were seeded onto 48-well plates and incubated at 37°C overnight. Then, 0.25 μg of *in vitro*-transcribed RNA was transfected using the TransIT-mRNA Transfection kit (Mirus) according to the manufacturer**’**s protocol.

### Quantitative RT-PCR

Quantitative RT-PCR was performed with 0.25μg of total RNAs by using QIAGEN One step PrimeScript RT-PCR Kit (Cat: RR064A) according to the manufacturer’s protocol. Serially diluted *in vitro*-transcribed EV71 RNAs were used as standard templates. Primers and probe used to detect EV71 VP1 gene are as follows: Forward primer: CAA TCA TGC TCT CGT CAC TAG C, reverse primer: CAC ACA GGT GAG CAG TCA TCG, probe: FAM-ACA GGC AAG GTT CCA GCA CTC CAA GC-BHQ1. The PCR reaction started at 42°C for 10 min, and 95°C for 30 s before cycling 5 reactions of 95°C for 10 s/55°C for 10 s/72°C for 15 s, followed by cycling 40 reactions of 95°C for 10 s/60°C for 40 s. The fluorescence signal was collected at 60°C at the last step.

### Luciferase activity

Cells were washed once with PBS and lysed with 1 × passive lysis buffer (Promega). Luciferase activity was measured with Renilla luciferase substrate (Promega) according to the manufacturer’s protocol. Luciferase activity was measured by Progema GloMaX 20/20 Luminometer.

### Virus inactivation by UV

The inactivated virus was obtained by ultraviolet radiation for one hour on ice performed in a cell culture hood. The UV-inactivation efficacy was determined by infecting RD cells with the UV treated virus and quantification of the viral RNA by real-time PCR or by infecting RD cells with UV-treated EV71-Nluc reporter virus and quantification of the luciferase activity. After this treatment, no obvious CPE and no viral RNA amplification were observed.

### Plague assay

Virus stocks and samples were titred by infection of Vero cells with 10-fold serial dilutions in DMEM with 2% FBS. Two hundred millilitre of diluted virus was added to each 6 well plates and after 1 h of infection, the well was overlaid with 0.6% agarose in MEM supplemented with 4% FBS. Plaques were enumerated by crystal violet staining after 72 h.

### Western blotting

After discarding the cell culture medium and washing with PBS, cells were lysed using 2 × SDS buffer (Including 100 mM Tris-Cl [pH 6.8], 4% SDS, 0.2% bromophenol blue, 20% glycerol, 10% 2-mercaptoethanol). The lysis solution was collected and boiled for 10 min. Protein was separated by SDS-PAGE and transferred to PDVF membrane. The membranes incubated with blocking buffer (PBS, 5% milk, 0.05% Tween) for 1 h and then washed with PBS-T (PBS, 0.05% Tween) and incubated with primary antibody(Mouse Anti- Enterovirus71 VP1 Abcam, cat: ab169442 from Abcam company) diluted in the blocking buffer by1:1000 at 4°C overnight. After three washes with PBS-T, the membrane was incubated with secondary antibody (Goat anti-mouse IgG-HRP, cat: sc-2005 from American Santa Cruz Biotechnology). After three washes with PBS-T, the membrane was visualized by Western Lightning Plus-ECL substrate (PerkinElmer, NEL10500).

### Immunofluorescence and microscopy

Cells were fixed with 4% paraformaldehyde in PBS for 10 min at room temperature. After washing with PBS for three times, the cells were incubated with 0.2% triton-X100 and 3.0% BSA diluted by PBS. Then cells were incubated using mouse anti-enterovirus71 antibody (MAB979, Merck) diluted by 1:1000 using 3.0% BSA at 4°C overnight. After washing for three times by PBS, cells were incubated using Alexa Fluor 488 conjugated donkey anti-mouse IgG antibody (CA21202S, Thermo Fisher Scientific) diluted by 1:500 using 3.0% BSA. After washing for three times, cells were incubated with DAPI for 5 min before observed with a fluorescence microscopy.

### Infection of animal

Three-day-old suckling mice (2.0–2.2 g) were injected with EV71 at a dosage of 1.4 × 10^4^ pfu/mouse diluted in 50μl PBS through intraperitoneal (i.p.) injection. Or the mice were infected with 50μl PBS (Mock). The body weight of mice and the survival proportions of mice were recorded at the same time every day after infection. The sickness of mice was evaluated using a graded score as reported before as follows: 0, healthy; 1, slow movement; 2, weakness in hind limbs; 3, paralysis in single limb; 4, paralysis in two limbs; and 5, death. On day 5 post infection, when the sickness of mice matched the third clinical graded score, mice were sacrificed and different tissues were collected.

Routine haematoxylin and eosin (H&E) staining of various mouse tissues was performed using 3.7% formaldehyde-fixed and paraffin-embedded sections (4 μm). To quantify viral RNAs in tissues, tissues were placed in tissue homogenization tubes (Biospec Products Inc., Bartlesville, OK) with a mixture of homogenization beads and 500 μl of Trizol reagent. Tubes were homogenized using a Tissuelyser-24 machine (Shanghai Jingxin Industrial Development Co., Ltd.) for 3.5 oscillations/min (1000) in 1-min intervals. RNAs were extracted and dissolved in RNase-free water and quantified by determining the A260 absorbance and used for q-PCR.

## Results

### Construction and characterization of the infectious clone of a clinical EV71 isolate

**Figure 1.**
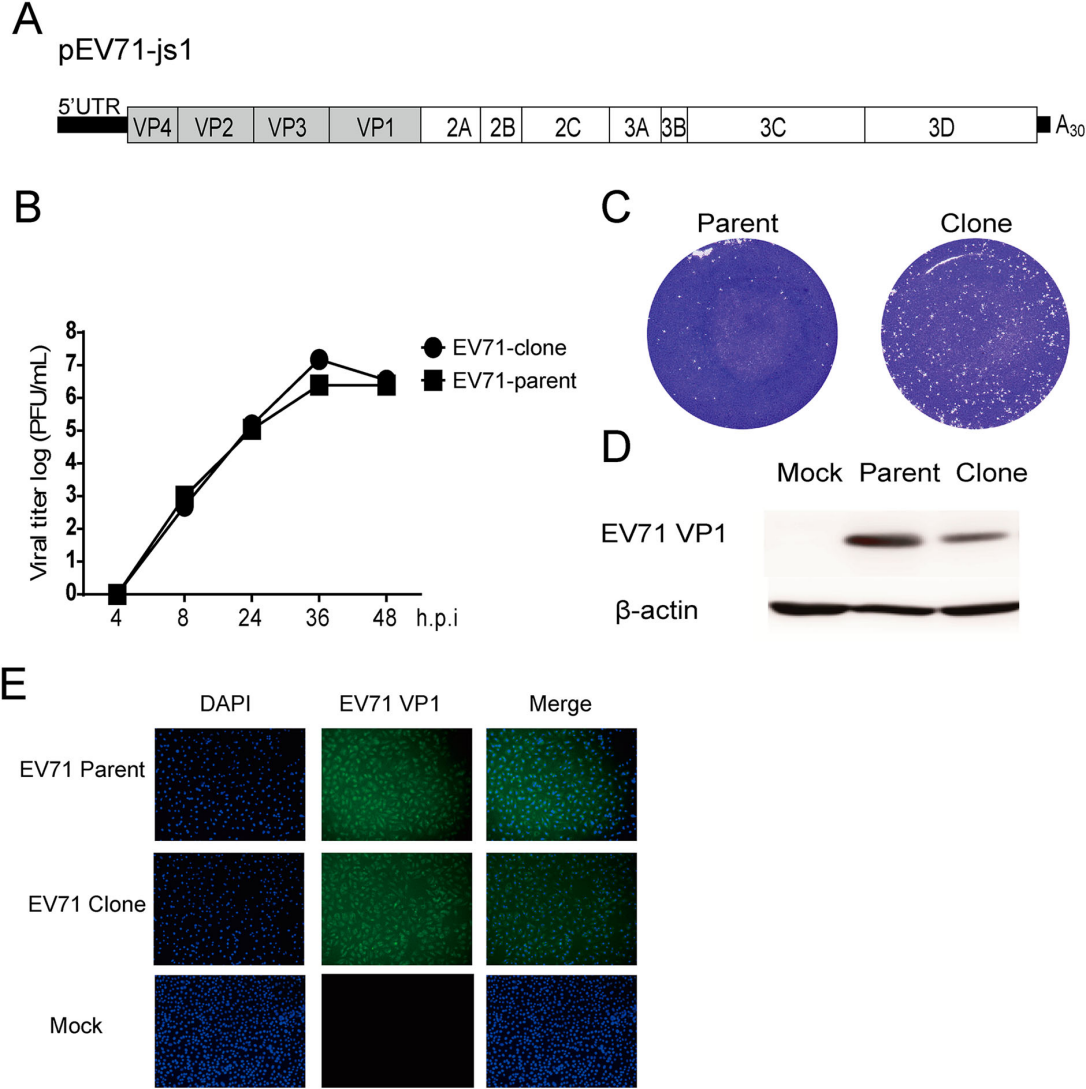
Construction and characterization of infectious clone-derived EV71. (A) Scheme of the cDNA constructs of EV71. A T7 promoter was flanked to the 5′ of the viral genome and a ployA (A30) was added to the 3′ of the viral genome. A Hind III site was added behind the polyA sequence. (B–D) RD cells were infected with EV71 695F virus stock (EV71-parent) and infectious clone-derived EV71 (EV71-clone) at MOI of 0.1. (B) At the indicated time points, the supernatants were collected and the virus titre was determined in Vero cells and plotted (*n* = 2). (C) Representative plaque morphology. (D) Representative infected cell lysates were analyzed by western blotting with anti-EV71 VP1 and anti-β-actin antibodies. (E) Vero cells were infected with EV71 695F virus stock (EV71-parent) and infectious clone-derived EV71 (EV71-clone) at MOI of 0.1. At 1 days post infection, the cells were fixed and immune-stained with anti-EV71 VP1 antibody, after counterstain-stained with DAPI and observed with confocal microscopy.

Previously, we isolated an EV71 strain from a severe HFMD patient and the virus was known to cause paralysis and death symptoms in ICR neonatal mice [21]. However, the virulence of the virus is unstable during passage on cell culture, leading to lower mortality rate and losing paralysis phenotype in mice (unpublished data). We reasoned that cell-culture derived mutations during virus passage might attenuate the viral virulence. When sequenced the virus VP1 gene and found a glutamic acid (E) to glycine (G) mutation at the residue 145 of VP1, even after two-rounds of virus passage (data not shown). This residue has been demonstrated to be a determinant of viral virulence [20, 22] probably by acting as a molecular “switch” to control the binding of the virus to PSGL-1 receptor [23]. To prevent this mutation, as well as excluding mutations at other sites, we sought to construct the cDNA clone of the isolated virus. Vero cells were infected with the original isolated stock (695F C+1). Viral RNAs were reversely transcribed. Four fragments of the cDNAs covering the whole genome of EV71 with overlapped restriction enzyme sites were amplified, and cloned into pANCR vector stepwise by restriction enzyme digestion. The finally assembled plasmid was named as pEV71-js1 (Figure 1(A)). When we transfected the *in-vitro*-transcribed viral RNAs into Vero cells, the rescued virus was infectious. Compared to the parental virus stock (parent), the cDNA clone-rescued (clone) virus showed similar growth kinetics in RD cells (Figure 1(B)) and similar plague morphology (Figure 1(C)). The viral replication was further verified by western blotting with anti-EV71 VP1 antibody (Figure 1(D)) and immunostaining of the virally infected cells with anti-EV71 VP1 antibody (Figure 1(E)). Thus, we successfully generated the infectious cDNA clone of an EV71 isolate.

### Infection of different neonatal mouse strains with the infectious clone-derived EV71

**Figure 2.**
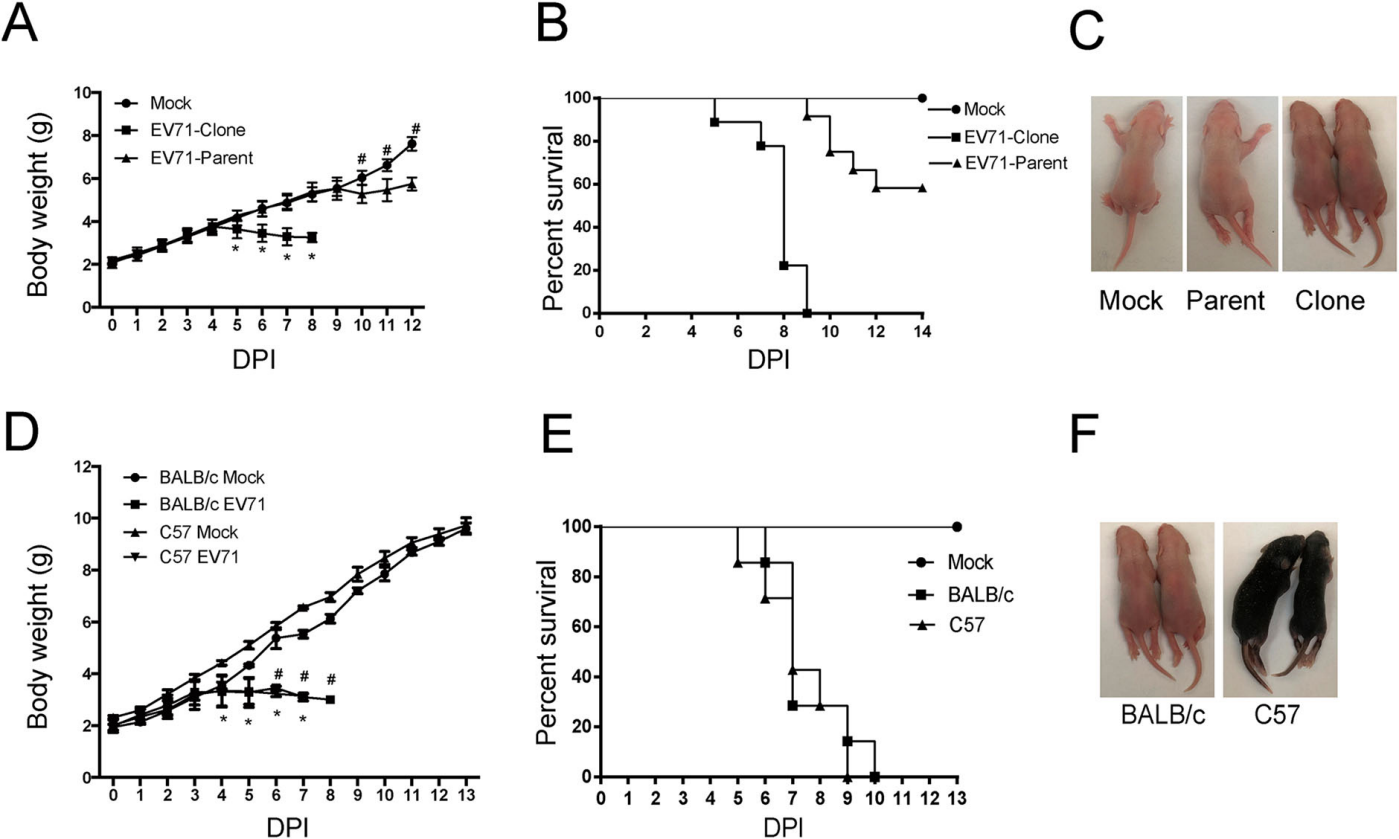
Infection of mouse with infectious clone-derived EV71. (A–C) Three-day-old ICR mice were infected with EV71 695F virus stock (EV71-parent) and infectious clone-derived EV71 (EV71-clone) at a dosage of 1.4 × 10^4^ pfu per mouse by intraperitoneal (i.p.) injection. Or the mice were infected with PBS (Mock). (A) The body weight of mice in different days after infection. All mice dead in EV71-Clone group from 9 days post infection (Mean ± SD, *n* = 10; *EV71-Clone and EV71 parent *P* < 0.01; # Mock and EV71 parent *P* < 0.01; two-tailed, unpaired *t*-test). (B) The mortality of mice (*n* = 10). (C) Representative paralysis symptoms of foetal mice at day 5 post infection. (D–F) Three-day-old Balb/C and C57 mice were infected with infectious clone-derived EV71 at a dosage of 1.4 × 10^4^ pfu per mouse by intraperitoneal (i.p.) injection as described above. (D) The body weight of mice in different days after infecting EV71-clones. All mice dead in C57 group from 8 days post infection. All mice dead in BALB/c group from 9 days post infection (Mean ± SD, *n* = 10, #BALB/c *P* < 0.01; *C57 *P* < 0.01; two-tailed, unpaired *t*-test). (E) The mortality of mice (*n* = 10). (F) Representative paralysis symptoms of foetal mice at day 5 post infection.

**Figure 3.**
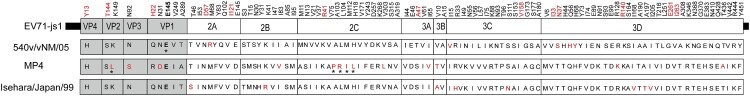
Amino acid sequence alignment of the open reading frames of EV71 strains. The amino acid sequence of 540v/vNM/05 (accession number, JQ965759), MP4 (accession number, JN544419) and Isehara/Japan/99 (accession number, LC375764) and EV71-js1 were analyzed by MegAlign (DNAstar) and the amino acid variations were denoted. Unique amino acid variations are in red. The asterisks indicate the modified or mouse-adapted variations. The E145 in VP1 are in bold.

Then we evaluated the infectivity of the cDNA clone-derived virus on mice. Our previous study has shown that the parental stock (EV71, 695F) can infect ICR suckling mouse and cause paralysis and death [21]. We infected 3-day-old ICR mice with the parental stock (parent) and the virus rescued from the cDNA clone (clone) by intraperitoneal injection (i.p.) at a dose of 1.4 × 10^4^ pfu/per mouse. On day 5 post infection, the mice infected with virus rescued from the cDNA clone (clone) began to lose weight whereas the mice infected with the parental stock (parent) didn’t start to lose weight until day 10 post infection (Figure 2(A)). Within two weeks, the mortality rate of mice infected with the virus rescued from the cDNA clone (clone) was 100% whereas the mice infected with the parental stock (parent) was 58.3% (Figure 2(B)). Accordingly, mice infected with virus rescued from the cDNA clone (clone) developed symptoms of lower limb paralysis on day 5 post infection, whereas mice infected with the parental stock (parent) developed symptoms on day 10 post infection (Figure 3(C)). These results suggest more virulence of the virus rescued from the cDNA clone than the parental virus. We speculated that the virus rescued from the cDNA clone has homogenous viral genome while the parental virus stock may have heterogeneous viral genomes containing mutations such as VP1.145G that attenuates the viral virulence. We also infected 3-day-old ICR mice with the cDNA clone-derived virus at the same dosage by oral route and got similar phenotypes as by intraperitoneal injection (Supplementary Figure 1). As oral administration was more traumatogenic to neonatal mice (data not shown and supplementary Figure 1B), we used intraperitoneal injection (i.p.) in the following study.

We also tested whether the cDNA-rescued virus infected other mouse strains or not. Similarly, we infected 3-day-old Balb/c and C57 mice with cDNA-rescued virus as described above. As in the ICR mice, viral infection caused weight loss from 5 days post infection (Figure 2(D)), 100% mortality rate within 2 weeks (Figure 2(E)) and lower limb paralysis (Figure 2(F)) in the infected mice.

### Sequence analysis of EV71-js1

We compared the amino acid sequence of the EV71-js1 with the sequences of three reported mouse-virulent EV71 strains 540v/vNM/05, MP4 and Isehara/Japan/99 that are capable of infecting neonatal mice or adult hSCARB2-transgenic mice [24]. The 540v/vNM/05 strain was modified by mutating the Q145 of VP1 to E145 according to a mouse-adapted strain MP-26M [25]. MP4 is a mouse-adapted strain [17] whereas Isehara/Japan/99 is a non-adapted strain that can infect hSCARB2-transgenic mice [12]. Comparing with these sequences, there are unique genetic variations in EV71-js1 in the VP4 (Y13), VP2 (T144), VP1 (H22), 2A (D57, I121), 2C (R41), 3A (A47), 3C (V158) and 3D (I33, N37, R140, E261, I263) regions (Figure 3 and Supplementary Figure 2). All the sequences have the Glutamic acid (E) at the position 145 in VP1 (Figure 3).

### Infectivity of the EV71 bearing a single cell-culture-derived mutation

**Figure 4.**
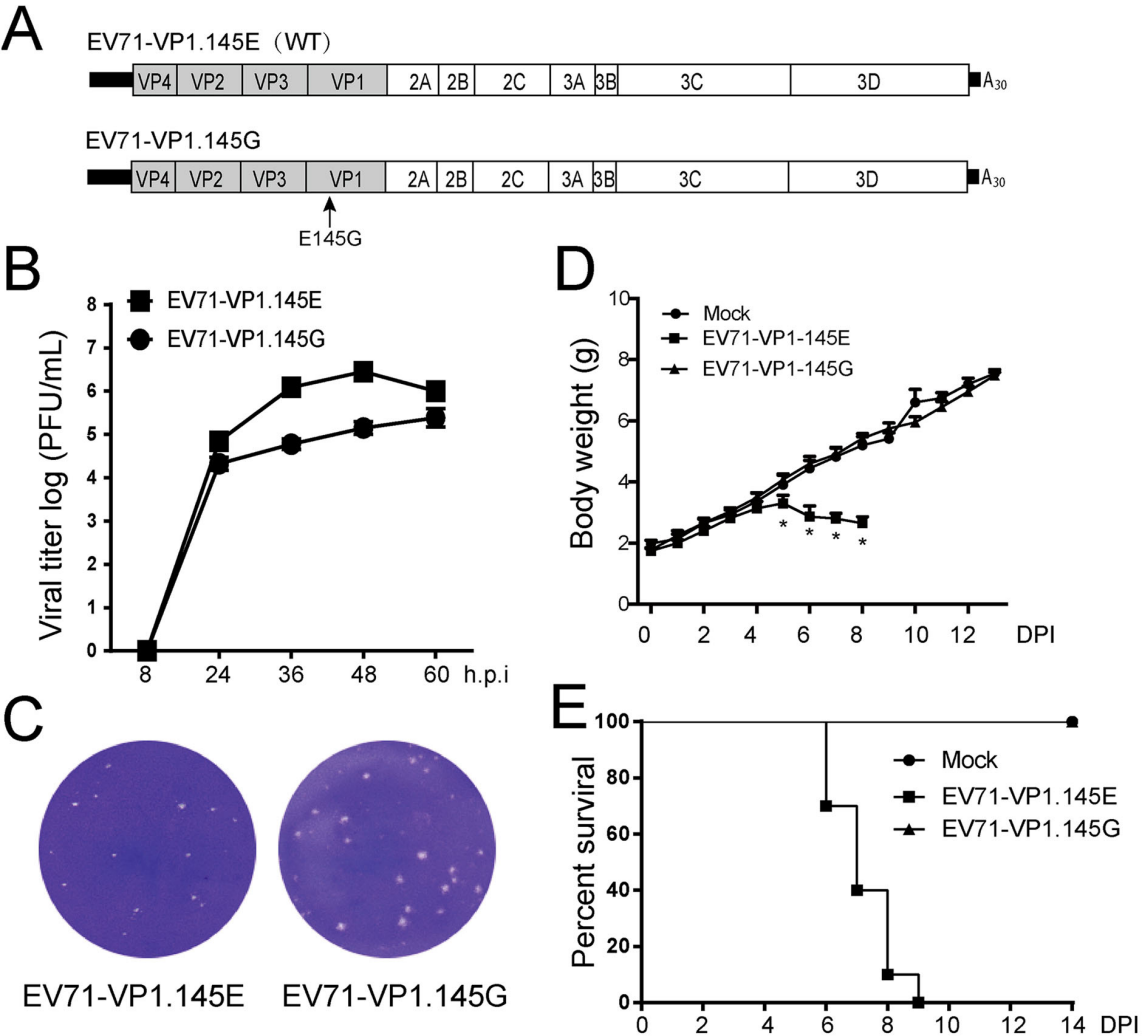
Infectivity of the EV71 bearing a single cell-culture-derived mutation. (A) Scheme of the cDNA constructs of EV71 and mutants. There is a glutamic acid at residue 145 of VP1 in the wild type (WT) sequence whereas a glycine at the same residue in the mutated sequence (Mutant). (B) The growth curves of EV71-VP1.145E and EV71-VP1.145G viruses. Vero cells were infected with viruses at MOI of 0.1. The supernatants were collected at indicated time points and the viral titres were determined by plague assay at duplicates in Vero cells and the titres were plotted (*n* = 2). (C) The plaque morphology of EV71-VP1.145E and EV71-VP1.145G virus in the infected Vero cells. (D–E) Three-day-old ICR mice were infected with infectious clone-derived EV71-VP1.145E and EV71-VP1.145G at a dosage of 1.4 × 10^4^ pfu per mouse by intraperitoneal (i.p.) injection as described above. (D) The body weight of mice in different days after infection. All mice dead in EV71-VP1.145E group from 9 days post infection. (Mean ± SD, *n* = 10. *EV71-VP1.145E and EV71-VP1.145G *P* < 0.01; two-tailed, unpaired *t*-test). (E) The mortality of mice (*n* = 10).

In order to explore if the less virulence of the parental stock is due to the cell-culture-adapted mutation E145G on VP1, we introduced the E145G mutation on the cDNA clones (Figure 4(A)) and rescued virus bearing this mutation. We first compared the growth kinetics of the mutated virus (VP1.145G) with the wild-type virus (VP1.145E). The mutated virus exhibited slower growth kinetics (Figure 4(B)) and blurry plague morphology (Figure 4(C)). In contrast to the wild-type virus, the mutated virus did not cause weight loss or mortality in the infected ICR mice (Figure 4(D, E)). Thus, the single cell-culture-derived mutation E145G in VP1 determines viral virulence in our mouse model.

### E145g mutation in VP1 reduces virus spread in cell lines

**Figure 5.**
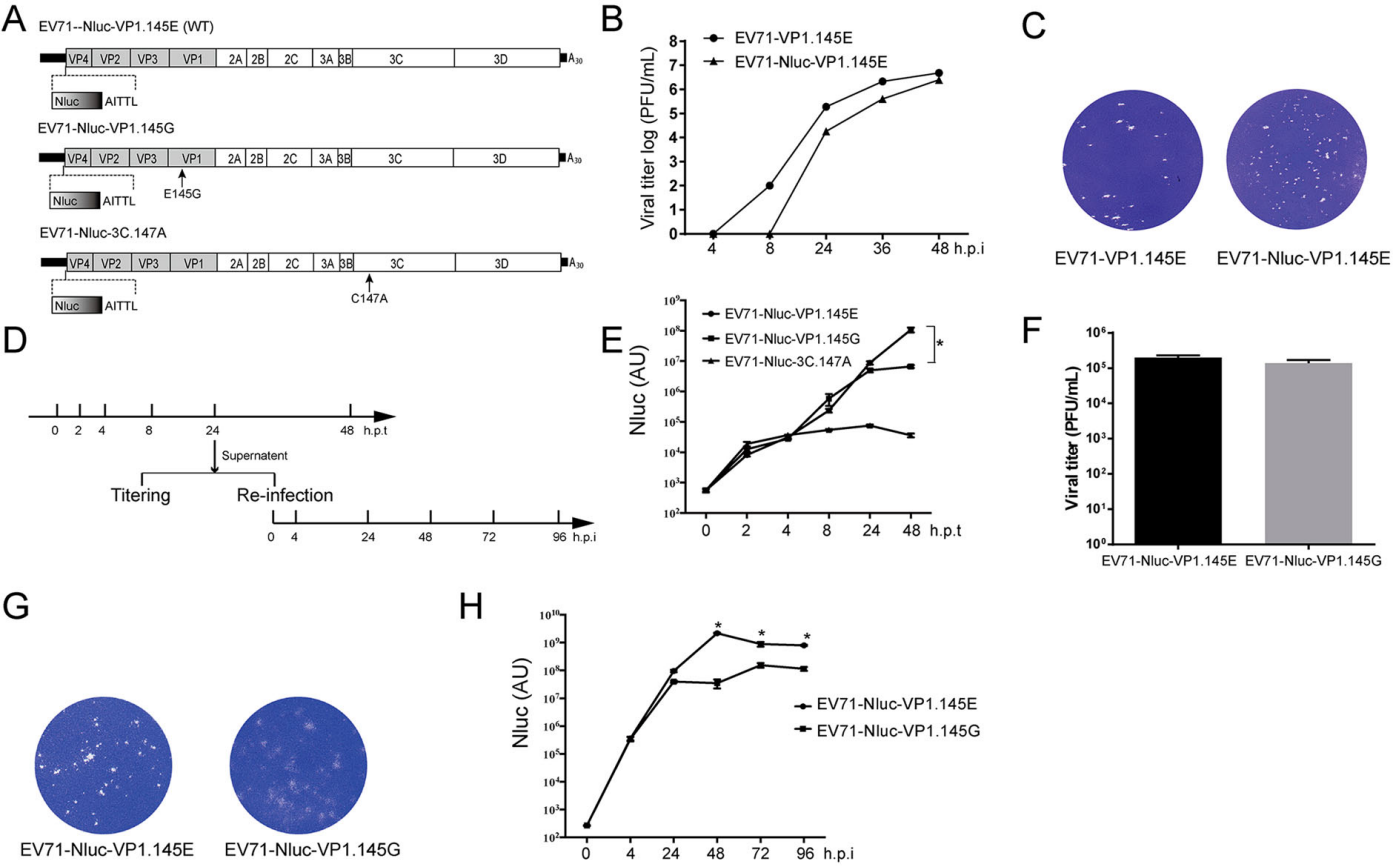
Characterization of EV71.VP1.145G virus. (A) Scheme of the cDNA constructs of EV71.Nluc reporter virus. Nluc, Nanoluciferase. The wild type (WT, 145E) and the mutant (145G) residue 145 of VP1 are shown. The 3C protease inactivated mutation C147A is shown. (B-C) The growth curves of EV71.Nluc reporter virus. (B) Vero cells were infected with EV71 (EV71-VP1.145E) and EV71-Nluc (EV71-Nluc-VP1.145E) viruses at a MOI of 0.1. At indicated time points, the viruses in the supernatants were tittered by plague assay (*n* = 2). (C) The plaque morphology of EV71-VP1.145E and EV71-Nluc-VP1.145E virus in infected Vero cells. (D) Schematic of the experiment design for E to H. The RNAs of EV71-Nluc-VP1.145E, EV71-Nluc-VP1.145G and EV71-Nluc-3C-147A were transfected into Vero cells. The supernatants were collected at the indicated time points and the luciferase activity was determined. The virus tittering in the supernatants collected at 24 h post transfection (h.p.t) were determined. And the supernatants were used to re-infect naïve Vero cells, and at various time points, the luciferase activity in the supernatants of the re-infected cells were determined. (E) Luciferase activity in the supernatants at various time points after transfection (Mean ± SD, *n* = 3). AU, arbitrary light units. (F) Virus titres in the supernatants collected at 24 h post transfection. (*n* = 2). (G) Representative plaque morphology of EV71-Nluc-VP1.145E and EV71-Nluc-VP1.145G. (H) Luciferase activity in the supernatants of the re-infected cells (Mean ± SD, *n* = 3). AU, arbitrary light units. From E to H, similar results were obtained at another independent experiment.

We further explored why the E145G attenuates the virulence. To facilitate monitoring the viral replication signal, we generated a cDNA clone with a Nanoluc reporter (Figure 5(A)). Compared to the virus without reporter gene, the EV71-Nluc reporter virus exhibited slower growth kinetics (Figure 5(B)) but similar plague morphology (Figure 5(C)). We then introduced the E145G mutation into the EV71-Nluc reporter clone. We also introduced a point mutation C147A in the 3C protease to inactivate the protease (Figure 5(A)). To monitor the effect of the E145G mutation on the viral life cycle, we transfected the *in-vitro*-transcribed viral RNAs into cells and measured the intracellular luciferase activity at various time points post transfection (Figure 5(D)). Upon transfection of EV71-Nluc-3C.147A RNA, we could only detect luciferase activity as early as 2 h post transfection and the luciferase signal didn’t increase at the following time points. This luciferase activity reflects the translation of the incoming viral RNA without viral genome amplification due to the 3C protease inactivation (Figure 5(E)). Transfection of EV71-Nluc-VP1.145E RNA and EV71-Nluc-VP1.145G RNA resulted in similar luciferase signal before 24 h (Figure 5(E)). However, there were significantly lower luciferase signal in the EV71-Nluc-VP1.145G RNA-transfected cells than that in the EV71-Nluc-VP1.145E RNA-transfected cells (Figure 5(E)). These results suggest an effect of the E145G mutation on the late step of viral life cycle. We then collected the supernatants from the EV71-Nluc-VP1.145E RNA- and EV71-Nluc-VP1.145G RNA-transfected cells at 24 h post transfection when they had similar luciferase signals (Figure 5(D)) and then determined the virus titre in the supernatants by plague assay. There were similar virus titres for these two viruses (Figure 5(F)). However, the virus from the EV71-Nluc-VP1.145G RNA-transfected cells exhibited blurry plague morphology (Figure 5(G)). We then infected naïve Vero cells with these supernatants and measured the intracellular luciferase activity at various time points post infection. The luciferase activity was similar at 4 h post infection (Figure 5(H)), suggesting no difference of virus entry between these two viruses. From 24 h post infection, the luciferase signal in the EV71-Nluc-VP1.145G infected cells was significantly lower than that in the EV71-Nluc-VP1.145E infected cells (Figure 5(H)). It was noted that the luciferase signal of EV71-Nluc-VP1.145E decreased from 48 h post infection due to cytopathic effect (Figure 5(H)). Taken together, these data suggest that the E145G mutation in VP1 reduces virus spread in cultured cells.

### Pathology of EV71-infected mice

**Figure 6.**
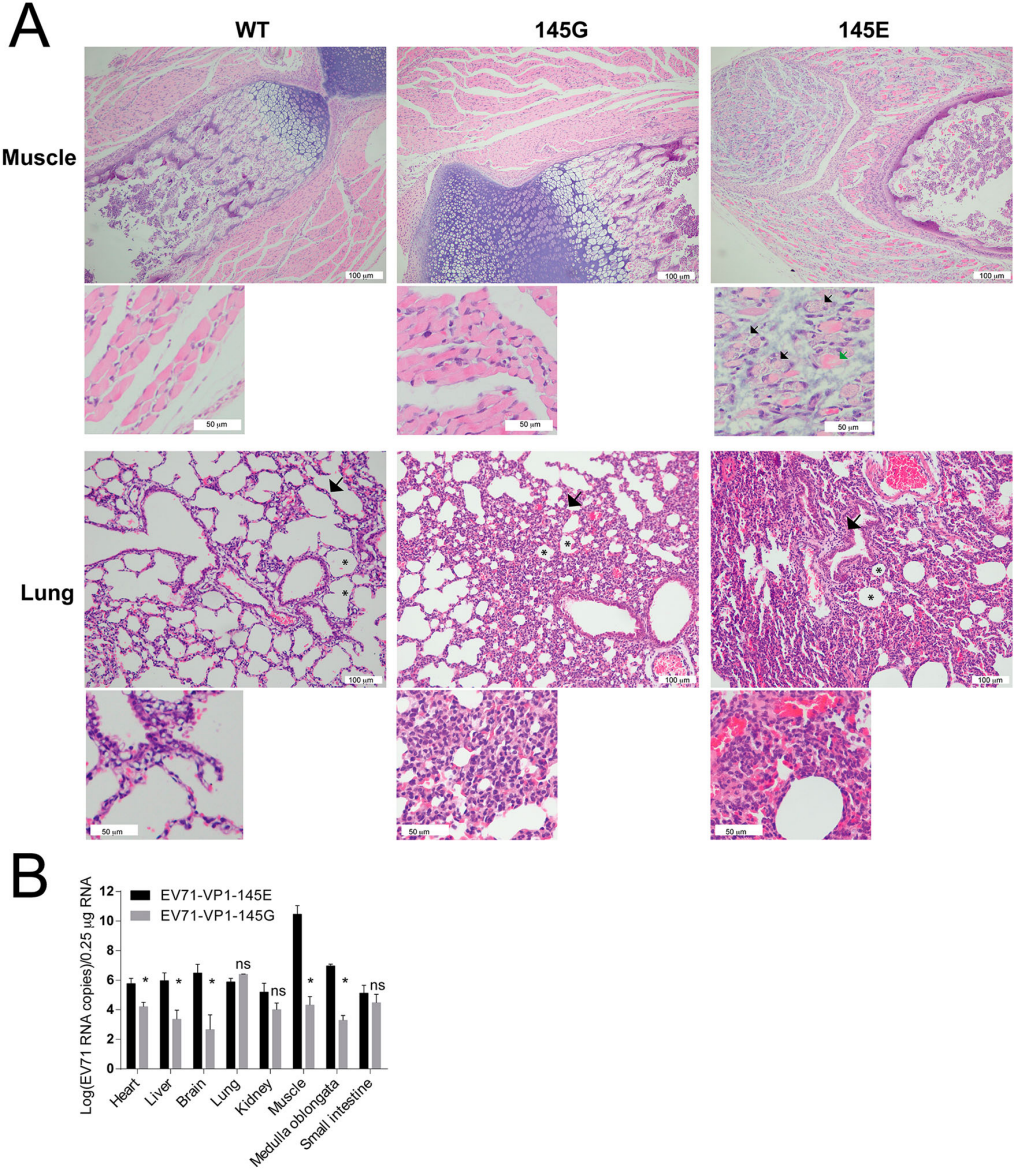
Pathology of EV71-infected mice. (A) ICR mice were infected with EV71-VP1.145E and EV71-VP1.145G as described above. Mice were sacrificed on day 5 post infection when the sickness of mice matched the third clinical graded score (see material and methods). Different tissues were fixed and paraffin-embedded sections were performed haematoxylin and eosin (H&E) staining. White scale bars are shown. In the muscle tissue, the black arrows indicate the myolysis; the green arrow indicates cells without complete myolysis. In the Lung tissue, the arrows indicate widened alveolar septum; the asterisks indicate diminished alveolar spaces. (B) Tissue tropism of EV71. Viral RNAs in different tissues were quantified by quantitative real-time PCR (Mean ± SD, *n* = 5) (*P < 0.05; two-tailed, unpaired *t*-test).

To explore the pathogenesis of EV71 infection, we infected ICR mice with EV71-VP1.145E and EV71-VP1.145G viruses, and examined the histologic changes of different tissues on day 5 post infection when the sickness of mice matched the third clinical graded score (see material and methods) and symptoms began to appear. The tissues were fixed, paraffin-embedded, sectioned and stained with Haematoxylin and eosin (HE). There were no obvious histologic changes in the brain, heart, liver, medullar oblongata in the EV71-VP1.145E and EV71-VP1.145G infected groups compared to the mock-infected group (Supplementary Figure 3). In contrast, EV71-VP1.145E infected mice exhibited massive myolysis (Figure 6(A), arrow), glomerular atrophy (Supplementary Figure 3), which was not observed in the EV71-VP1.145G infected mice or in the mock infected mice (Figure 6(A)). Both EV71-VP1.145E and EV71-VP1.145G infected mice exhibited villous blunting in the small intestine (Supplementary Figure 3). Strikingly, we observed widened alveolar septum (Figure 6(A), arrows), diminished alveolar spaces (Figure 6(A), asterisk) and lymphocytes infiltration into the lung in the EV71-VP1.145E and EV71-VP1.145G infected mice (Figure 6(A)), with more severe phenotypes in the EV71-VP1.145E infected mice (Figure 6(A)). Viral RNAs in different tissues were determined by quantitative RT-PCR. Viral RNAs were detected in the heart, liver, brain, lung, kidney, leg skeletal muscle, medulla oblongata and small intestine tissues in both EV71-VP1.145E and EV71-VP1.145G infected mice. However, the viral RNA levels in the heart, liver, brain, leg skeletal muscle and medulla oblongata tissues in the EV71-VP1.145E infected group were significantly higher than that in the EV71-VP1.145G infected group (Figure 6(B)). Strikingly, there was much higher viral RNA level in the leg skeletal muscle than that in other tissues in the EV71-VP1.145E infected mice (Figure 6(B)). These results demonstrate that EV71-VP1.145E virus spread to different tissues and replicate mostly in the skeletal muscle tissue.

### Viral dynamics in EV71-infected mice

**Figure 7.**
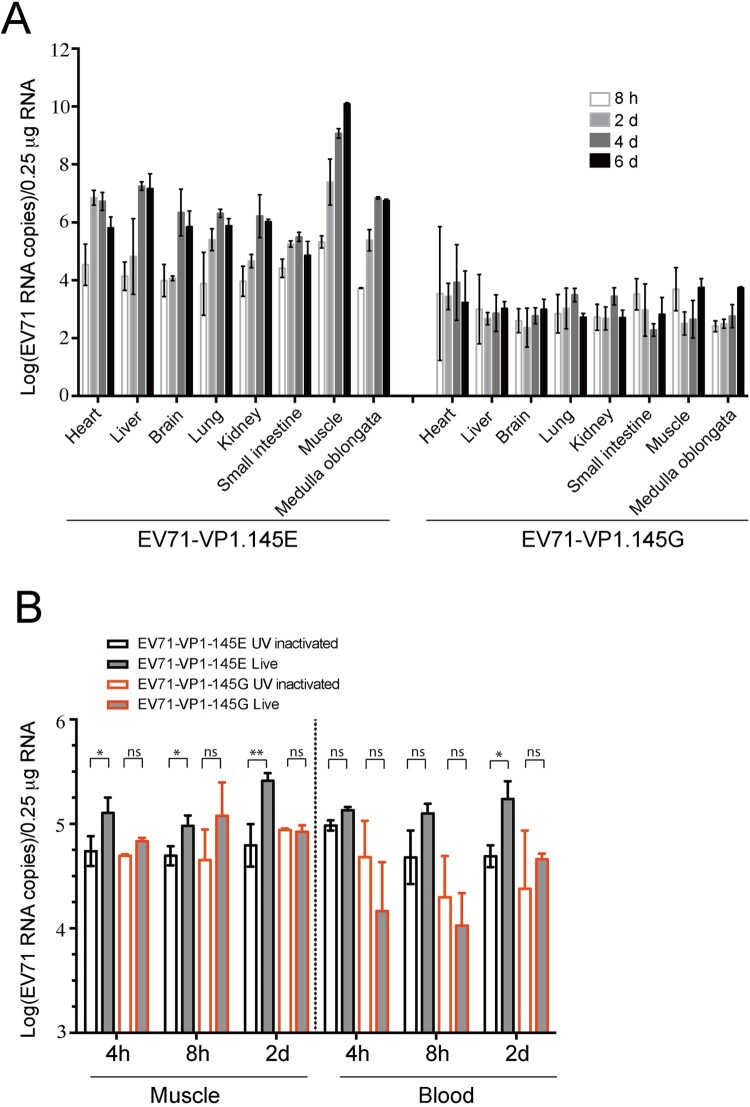
Viral dynamics in EV71-infected mice. (A) ICR foetal mice were infected with EV71-VP1.145E and EV71-VP1.145G viruses as described above and sacrificed at various time points after infection. Different tissues were isolated and the viral levels were determined by qPCR (Mean ± SD, *n* = 5). (B) ICR foetal mice were infected with EV71-VP1.145E and EV71-VP1.145G viruses or UV-inactivated counterparts. At indicated time points after infection, the viral RNA levels in blood and leg skeletal muscle were determined (Mean ± SD, *n* = 5) (*P < 0.05; **P < 0.01; two-tailed, unpaired *t*-test).

We tracked the virus during the infection by quantifying the viral RNAs in different tissues at various time points post infection. We infected ICR mice with EV71-VP1.145E and EV71-VP1.145G viruses, collected different tissues at 8 h, 2, 4 and 6 days post infection, and extracted total RNAs from the tissues and quantified the viral RNAs by quantitative RT-PCR. In the EV71-VP1.145E virus-infected mice, increasing viral RNA level in the heart, liver, brain, lung, kidney, leg skeletal muscle, medulla oblongata and small intestine were detected during the infection course, with highest viral RNA level in the leg skeletal muscle (Figure 7(A)). It was noted that viral RNA levels increased dramatically at 2 days post infection in the leg skeletal muscle, heart, lung and medulla oblongata tissues, compared to the viral RNA levels at 8h post infection. However, the dramatic increase of viral RNA levels occurred in the liver, brain and kidney tissues only until 4 days post infection, which suggests that the viral replication rate in the leg skeletal muscle, heart, lung and medulla oblongata precedes that in liver, brain and kidney. In contrast to the EV71-VP1.145E virus-infected mice, in the EV71-VP1.145G virus-infected mice, no obvious increase of RNA level in the examined tissues was detected (Figure 7(A)), suggesting no spread of virus.

To elucidate the possible primary site for viral replication, we infected ICR mice with the active and UV-inactivated EV71-VP1-145G and EV71-VP1-145E virus, and then tracked the viral RNA level in the leg skeletal muscle and blood at early time points (4h, 8h and 2d) post infection. We used the UV-inactivated virus to segregate the authentic viral replication from the input viral RNA as the UV-inactivated virus did not replicate in the cells (see material and methods and data not shown) and the viral RNA levels in the UV-inactivated virus infected tissues serve as a background of the input viral RNA. Compared to the UV-inactivated virus-infected mice, the EV71-VP1-145E virus-infected mice had a significant increase of viral RNA level in leg skeletal muscle but not in the blood at 4h and 8h post infection (Figure 7(B)). Significant increase of viral RNA level was observed in both leg skeletal muscle and blood at 2d post infection (Figure 7(B)). These results suggest that viral replication in leg skeletal muscle precedes that in blood and the leg skeletal muscle tissue is likely the primary site for viral replication. In contrast to the EV71-VP1.145E virus-infected mice, in the EV71-VP1.145G virus-infected mice, we did not observe obvious increase of RNA level in leg skeletal muscle and blood, when comparing to the UV-inactivated virus (Figure 7(B)), suggesting no viral replication. Taken together, these data suggest that: (1) the muscle tissue is the primary site for viral amplification; (2) the VP1.E145G mutation did not result in virus spread.

## Discussion

EV71 is different from other HFMD-causing enteroviruses because EV71 can cause complications in the central nervous system, circulatory system, respiratory system and even death [26–28]. Limited studies have been performed to elucidate the pathogenesis of EV71-caused severe diseases mainly due to lack of simple animal models. Reported mouse models either require innate deficient [10, 11] or transgenic background [12, 13]. In addition, mouse-adapted strain [12, 14–17] or a high dose of virus is needed to establish the virus infection model [11, 18–20]. Reported mouse models cannot recapitulate similar symptoms observed in humans such as pulmonary oedema [29], an important phenotype of EV71 infection in humans.

In this study, we generated the infectious clone of a clinical isolate from a severe HFMD patient. When the virus rescued from the cDNA clone was administrated intraperitoneally (i.p.) to immune competent neonatal ICR, BALB/c and C57 mice at a low dosage of 1.4 × 10^4^ pfu per mouse, it caused the infected mice to have body weight loss, paralysis and death after 4–5 days of infection (Figure 2). In a mouse model infected with an EV71 mouse-adapted strain, the diseased mice exhibited histology changes in muscle, small intestine and kidney but not in the lung [17, 29], an important phenotype of EV71 infection in humans. In our model, the diseased mice exhibited similar phenotypes such as massive myolysis, glomerular atrophy, villous blunting in the small intestine (Figure 6(A)). Strikingly, in our model, we observed widened alveolar septum, diminished alveolar spaces and lymphocytes infiltration into the lung (Figure 6(A)). Similar phenotypes were only reported in EV71 rhesus monkey model [30].

It was noted that during the virus passage, a cell-culture derived point mutation E145G in the VP1 attenuated viral virulence in mice (Figure 4). Rescuing virus from cDNA clone can prevent the appearance of mutations that attenuate viral virulence, which may explain why we could infect the mice with a low dosage of 1.4 × 10^4^ pfu per mouse. It is possible that the 695F strain is a unique strain that is highly virulent in mice. EV71-js1 has unique genetic variations comparing to the sequence of other mouse virulent strains (Figure 3 and Supplementary Figure 2).

The E145 residue in VP1 has been demonstrated to be a determinant for viral virulence and E145G mutation abolishes viral virulence in mice [20, 22]. In the hSCARB2 transgenic mice model, EV71-VP1.145G can bind to heparan sulphate (HS) receptor and restrict the spread of virus in mice [20]. Consistent with these findings, we found the E145G mutation prevented virus spread within the tissues (Figures 6 and 7). We observed that virus with the E145G mutation exhibited slower replication kinetics and blurry plague morphology (Figure 4) and this might be due to the reduction of virus spread (Figure 5). It has been reported that the protein-protein interaction between the non-structural protein 2C and structural protein VP3 is required for enterovirus morphogenesis [3]. If the E145G mutation affects virion morphogenesis through a similar mechanism needs further studies.

EV71 infection mainly causes asymptomatic infection. In children, high titres of virus in blood disseminate to the skin and mucous membranes to causes HFMD. In a small portion of patients, EV71 infects the central nervous system (CNS) and causes severe disease [6]. The mechanism that EV71 infects the central nervous (CNS) is still poorly defined. By use of a mouse-adapted EV71 strain, it has been demonstrated that after oral inoculation, viral replication was first detected in the intestine and then in the limb muscles, spinal cord and then finally spread to the brain. The muscle is a major target for robust viral replication [17]. Similarly, in our model, when the virus was intraperitoneally administrated, skeletal muscle had the highest viral replication (Figure 7(A)). Increase of viral replication was first observed in the leg skeletal muscle, heart, lung and medulla oblongata tissues, and then in the liver, brain and kidney (Figure 7(A)). Compared to the UV-inactivated virus, viral replication was observed in leg skeletal muscle but not in the blood at very early time points after inoculation (Figure 7(B)), supporting the skeletal muscle as a primary site for initial viral replication in our model.

In summary, the EV71 mouse model described here does not need mouse with specific genetic background and mouse-adapted strains of virus. Rescuing virus from cDNA clone prevents the occurrence of mouse virulence-attenuated mutations. Thus, we provide a simple and stable mouse model for studying the pathogenesis of enterovirus-associated diseases and for developing anti-enterovirus drugs.

## Supplementary Material

Supplemental Material

## References

[1] Ho M, Chen ER, Hsu KH, et al. An epidemic of enterovirus 71 infection in Taiwan. Taiwan enterovirus epidemic working group. N Engl J Med. 1999 Sep 23;341(13):929–935. doi: 10.1056/NEJM199909233411301 10498487

[2] Liu Q, Huang X, Ku Z, et al. Characterization of enterovirus 71 capsids using subunit protein-specific polyclonal antibodies. J Virol Methods. 2013 Jan;187(1):127–131. doi: 10.1016/j.jviromet.2012.09.024 23046991

[3] Jiang P, Liu Y, Ma HC, et al. Picornavirus morphogenesis. Microbiol Mol Biol Rev. 2014 Sep;78(3):418–437. doi: 10.1128/MMBR.00012-14 25184560 PMC4187686

[4] Plevka P, Perera R, Cardosa J, et al. Crystal structure of human enterovirus 71. Science. 2012 Jun 8;336(6086):1274. doi: 10.1126/science.1218713 22383808 PMC3448362

[5] Zhou D, Zhao Y, Kotecha A, et al. Unexpected mode of engagement between enterovirus 71 and its receptor SCARB2. Nat Microbiol. 2019 Mar;4(3):414–419. doi: 10.1038/s41564-018-0319-z 30531980

[6] Cox JA, Hiscox JA, Solomon T, et al. Immunopathogenesis and virus-host interactions of enterovirus 71 in patients with hand, foot and mouth disease [Review]. Front Microbiol. 2017;8:2249. doi: 10.3389/fmicb.2017.02249 29238324 PMC5713468

[7] Chumakov M, Voroshilova M, Shindarov L, et al. Enterovirus 71 isolated from cases of epidemic poliomyelitis-like disease in Bulgaria. Arch Virol. 1979;60(3–4):329–340. doi: 10.1007/BF01317504 228639

[8] Hashimoto I, Hagiwara A, Kodama H. Neurovirulence in cynomolgus monkeys of enterovirus 71 isolated from a patient with hand, foot and mouth disease. Arch Virol. 1978;56(3):257–261. doi: 10.1007/BF01317855 205198

[9] Hashimoto I, Hagiwara A. Pathogenicity of a poliomyelitis-like disease in monkeys infected orally with enterovirus 71: a model for human infection. Neuropathol Appl Neurobiol. 1982 Mar-Apr;8(2):149–156. doi: 10.1111/j.1365-2990.1982.tb00269.x 7048123

[10] Chen H, Zhang Y, Yang E, et al. The effect of enterovirus 71 immunization on neuropathogenesis and protein expression profiles in the thalamus of infected rhesus neonates. Virology. 2012 Oct 25;432(2):417–426. doi: 10.1016/j.virol.2012.06.026 22819834

[11] Khong WX, Yan B, Yeo H, et al. A non-mouse-adapted enterovirus 71 (EV71) strain exhibits neurotropism, causing neurological manifestations in a novel mouse model of EV71 infection. J Virol. 2012 Feb;86(4):2121–2131. doi: 10.1128/JVI.06103-11 22130542 PMC3302383

[12] Fujii K, Nagata N, Sato Y, et al. Transgenic mouse model for the study of enterovirus 71 neuropathogenesis. Proc Natl Acad Sci U S A. 2013 Sep 3;110(36):14753–14758. doi: 10.1073/pnas.1217563110 23959904 PMC3767555

[13] Liu J, Dong W, Quan X, et al. Transgenic expression of human P-selectin glycoprotein ligand-1 is not sufficient for enterovirus 71 infection in mice. Arch Virol. 2012 Mar;157(3):539–543. doi: 10.1007/s00705-011-1198-2 22187102

[14] Huang SW, Wang YF, Yu CK, et al. Mutations in VP2 and VP1 capsid proteins increase infectivity and mouse lethality of enterovirus 71 by virus binding and RNA accumulation enhancement. Virology. 2012 Jan 5;422(1):132–143. doi: 10.1016/j.virol.2011.10.015 22078110

[15] Yu CK, Chen CC, Chen CL, et al. Neutralizing antibody provided protection against enterovirus type 71 lethal challenge in neonatal mice. J Biomed Sci. 2000 Nov-Dec;7(6):523–528. doi: 10.1007/BF02253368 11060501

[16] Yeh MT, Wang SW, Yu CK, et al. A single nucleotide in stem loop II of 5′-untranslated region contributes to virulence of enterovirus 71 in mice. PLoS One. 2011;6(11):e27082. doi: 10.1371/journal.pone.0027082 22069490 PMC3206083

[17] Wang YF, Chou CT, Lei HY, et al. A mouse-adapted enterovirus 71 strain causes neurological disease in mice after oral infection. J Virol. 2004 Aug;78(15):7916–7924. doi: 10.1128/JVI.78.15.7916-7924.2004 15254164 PMC446098

[18] Pan H, Yao X, Chen W, et al. Dissecting complicated viral spreading of enterovirus 71 using in situ bioorthogonal fluorescent labeling. Biomaterials. 2018 Oct;181:199–209. doi: 10.1016/j.biomaterials.2018.07.061 30086449

[19] Liao CC, Liou AT, Chang YS, et al. Immunodeficient mouse models with different disease profiles by in vivo infection with the same clinical isolate of enterovirus 71. J Virol. 2014 Nov;88(21):12485–12499. doi: 10.1128/JVI.00692-14 25142603 PMC4248922

[20] Kobayashi K, Sudaka Y, Takashino A, et al. Amino acid variation at VP1-145 of enterovirus 71 determines attachment receptor usage and neurovirulence in human scavenger receptor B2 transgenic mice. J Virol. 2018 Aug 1;92(15):e00681–18. doi: 10.1128/JVI.00681-18 29848584 PMC6052303

[21] Zhang X, Song Z, Qin B, et al. Rupintrivir is a promising candidate for treating severe cases of enterovirus-71 infection: evaluation of antiviral efficacy in a murine infection model. Antiviral Res. 2013 Mar;97(3):264–269. doi: 10.1016/j.antiviral.2012.12.029 23295352

[22] Kataoka C, Suzuki T, Kotani O, et al. The role of VP1 amino acid residue 145 of enterovirus 71 in viral fitness and pathogenesis in a cynomolgus monkey model. PLoS Pathog. 2015 Jul;11(7):e1005033. doi: 10.1371/journal.ppat.1005033 26181772 PMC4504482

[23] Nishimura Y, Lee H, Hafenstein S, et al. Enterovirus 71 binding to PSGL-1 on leukocytes: VP1-145 acts as a molecular switch to control receptor interaction. PLoS Pathog. 2013;9(7):e1003511. doi: 10.1371/journal.ppat.1003511 23935488 PMC3723564

[24] Shih C, Liao CC, Chang YS, et al. Immunocompetent and immunodeficient mouse models for enterovirus 71 pathogenesis and therapy. Viruses. 2018 Nov 28;10(12):674–693. doi: 10.3390/v10120674 30487421 PMC6316343

[25] Zaini Z, McMinn P. A single mutation in capsid protein VP1 (Q145E) of a genogroup C4 strain of human enterovirus 71 generates a mouse-virulent phenotype. J Gen Virol. 2012 Sep;93(9):1935–1940. doi: 10.1099/vir.0.043893-0 22647370

[26] Solomon T, Lewthwaite P, Perera D, et al. Virology, epidemiology, pathogenesis, and control of enterovirus 71. Lancet Infect Dis. 2010 Nov;10(11):778–790. doi: 10.1016/S1473-3099(10)70194-8 20961813

[27] Weng KF, Chen LL, Huang PN, et al. Neural pathogenesis of enterovirus 71 infection. Microbes Infect. 2010 Jul;12(7):505–510. doi: 10.1016/j.micinf.2010.03.006 20348010

[28] Liu SL, Pan H, Liu P, et al. Comparative epidemiology and virology of fatal and nonfatal cases of hand, foot and mouth disease in mainland China from 2008 to 2014. Rev Med Virol. 2015 Mar;25(2):115–128. doi: 10.1002/rmv.1827 25704797

[29] Chen CS, Yao YC, Lin SC, et al. Retrograde axonal transport: a major transmission route of enterovirus 71 in mice. J Virol. 2007 Sep;81(17):8996–9003. doi: 10.1128/JVI.00236-07 17567704 PMC1951457

[30] Zhang Y, Cui W, Liu L, et al. Pathogenesis study of enterovirus 71 infection in rhesus monkeys. Lab Invest. 2011 Sep;91(9):1337–1350. doi: 10.1038/labinvest.2011.82 21555996

