# Supplementary material for: An infectious clone of enterovirus 71(EV71) that is capable of infecting neonatal immune competent mice without adaptive mutations
Source: Emerg Microbes Infect. 2020 Feb 21;9(1):427–38. doi: 10.1080/22221751.2020.1729665 (PMC7048218; doi:10.1080/22221751.2020.1729665)
Supplement: Supplemental Material [file TEMI_A_1729665_SM5139.zip › Suppplementary_Figure_2R_final.docx]

MGSQVSTQRSGSYENSNSATEGSTINYTTINYYKDSYAATAGKQSLKQDP

DKFANPVKDIFTEMAAPLKSPSAEACGYSDRVAQLTIGNSTITTQEAANII

VGYGEWPSYCSDSDATAVDKPTRPDVSVNRFYTLDTKLWEKSSKGWYW

KFPDVLTETGVFGQNAQFHYLYRSGFCIHVQCNASKFHQGALLVAVLPE

YVIGTVAGGTGTEDTHPPYKQTQPGADGFELQHPYVLDAGIPISQLTVC

PHQWINLRTNNCATIIVPYINALPFDSALNHCNFGLLVVPISPLDYDQGA

TPVIPITITLAPMCSEFAGLRQAVTQGFPTELKPGTNQFLTTDDGVSAPIL

PNFHPTPCIHIPGEVRNLLELCQVETILEVNNVPTNATSLMERLRFPVSA

QAGKGELCAVFRADPGRNGPWQSTLLGQLCGYYTQWSGSLEVTFMFT

GSFMATGKMLIAYTPPGGPLPKDRATAMLGTHVIWDFGLQSSVTLVIPW

ISNTHYRAHARDGVFDYYTTGLVSIWYQTNYVVPIGAPNTAYIIALAAAQ

KNFTMKLCKDASDILQTGTIQGDRVADVIESSIGDSVSRALTHALPAPTG

QNTQVSSHRLDTGKVPALQAAEIGASSNASDESMIETRCVLNSHSTAETT

LDSFFSRAGLVGEIDLPLEGTTNPNGYANWDIDITGYAQMRRKVELFTY

MRFDAEFTFVACTPTGEVVPQLLQYMFVPPGAPKPDSRESLAWQTATNP

SVFVKLSDPPAQVSVPFMSPASAYQWFYDGYPTFGEHKQEKDLEYGACP

NNMMGTFSVRTVGTSKSKYPLVVRIYMRMKHVRAWIPRPMRNQNYLFK

ANPNYAGNSIKPTGASRTAITTLGKFGQQSGAIYVGNFRVVNRHLATHN

DWANLVWEDSSRDLLVSSTTAQGCDTIARCDCQTGVYYCNSMRKHYPV

SFSKPSLIYVEASEYYPARYQSHLMLAQGHSEPGDCGGILRCQHGVIGIV

STGGNGLVGFADVRDLLWLDEEAMEQGVSDYIKGLGDAFGTGFTDAVS

REVEALKNYLIGSEGAVEKILKNLIKLISALVIVIRSDYDMVTLTATLALIGC

HGSPWAWIKAKTASILGIPIAQKQSASWLKKFNDMANAAKGLEWVSNKI

SKFIDWLKEKIVPAAREKVEFLNNLKQLPLLENQISNLEQSAASQEDLEV

MFGNVSYLAHFCRKFQPLYATEAKRVYALEKRMNNYMQFKSKHRIEPVC

LIIRGSPGTGKSLATGIIARAIADKYHSSVYSLPPDPDHFDGYKQQVVTVM

DDLCQNPDGKDMSLFCQMVSTVDFIPPMASLEEKGVSFTSKFVIASTNA

SNIIVPTVSDSDAIRRRFYMDCDIEVTDSYKTDLGRLDAGRAAKLCSENN

TANFKRCSPLVCGKAIQLRDRKSKVRYSVDTVVSELIREYSNRSAIGNTIEA

LFQGPPKFRPIRISLEEKPAPDAISDLLASVDSEEVRQYCRDQGWIIPEAPT

NVERHLNRAVLVMQSITTVVAVVSLVYVIYKLFAGFQGAYSGAPKQVLKK

PALRTATVQGPSLDFALSLLRRNIRQVQTDQGHFTMLGVRDRLAVLPRH

SQPGKTIWIEHKLVNVLDAVELVDEQGVNLELTLITLDTNEKFRDITKFIP ENISTASDATLVINTEHMPSMFVPVGDVVQYGFLNLSGKPTHRTMMYNF

PTKAGQCGGVVTSVGKVVGIHIGGNGRQGFCAGLKRSYFASEQGEIQW

VKPNKETGRLNINGPTRTKLEPSVFHDIFEGNKEPAVLHSKDPRLEVDFE

QALFSKYVGNTLHEPDEYIKEAALHYANQLKQLEINTSQMSMEEACYGT

ENLEAIDLHTSAGYPYSALGIKKRDILDPTTRDVSRMKFYMDKYGLDLP

YSTYVKDELRSIDKIKKGKSRLIEASSLNDSVYLRMAFGHLYEAFHANPGT

ITGSAVGCNPDTFWSKLPILLPGSLFAFDYSGYDASLSPVWFRALELVLRE IGYSEEAISLIEGINHTHHVYRNKTYCVLGGMPSGCSGTSIFNSMINNIIIR

ALLIKTFKGIDLDELNMVAYGDDVLASYPFPIDCLELAKTGKEYGLTMTP

ADKSPCFNEVNWGNATFLKRGFLPDEQFPFLIHPTMPMREIHESIRWTK DARNTQDHVRSLCLLAWHNGKQEYEKFVSTIRSVPVGRALAIPNYENLR RNWLELF.
