# Supplementary figures and images for: An infectious clone of enterovirus 71(EV71) that is capable of infecting neonatal immune competent mice without adaptive mutations
Source: Emerg Microbes Infect. 2020 Feb 21;9(1):427–38. doi: 10.1080/22221751.2020.1729665 (PMC7048218; doi:10.1080/22221751.2020.1729665)

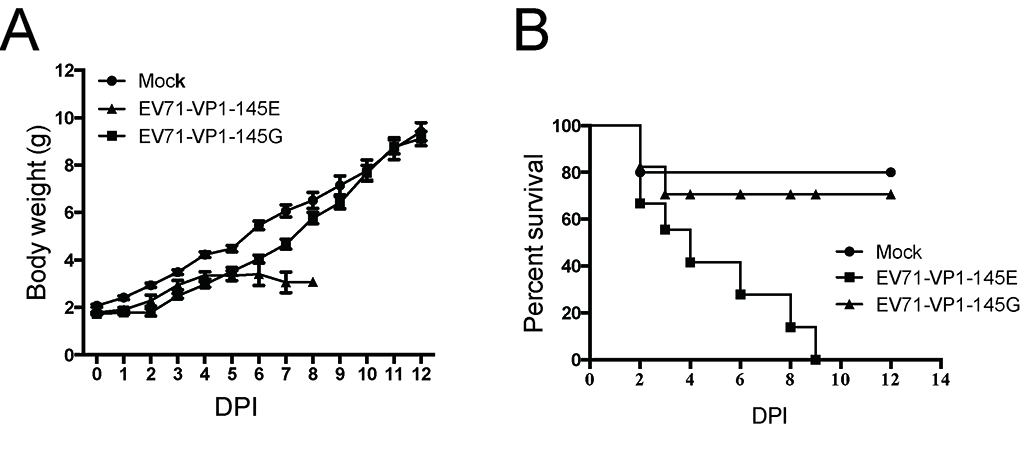

Supplement: Supplemental Material [file TEMI_A_1729665_SM5139.zip › supple Figure 1R.tif]

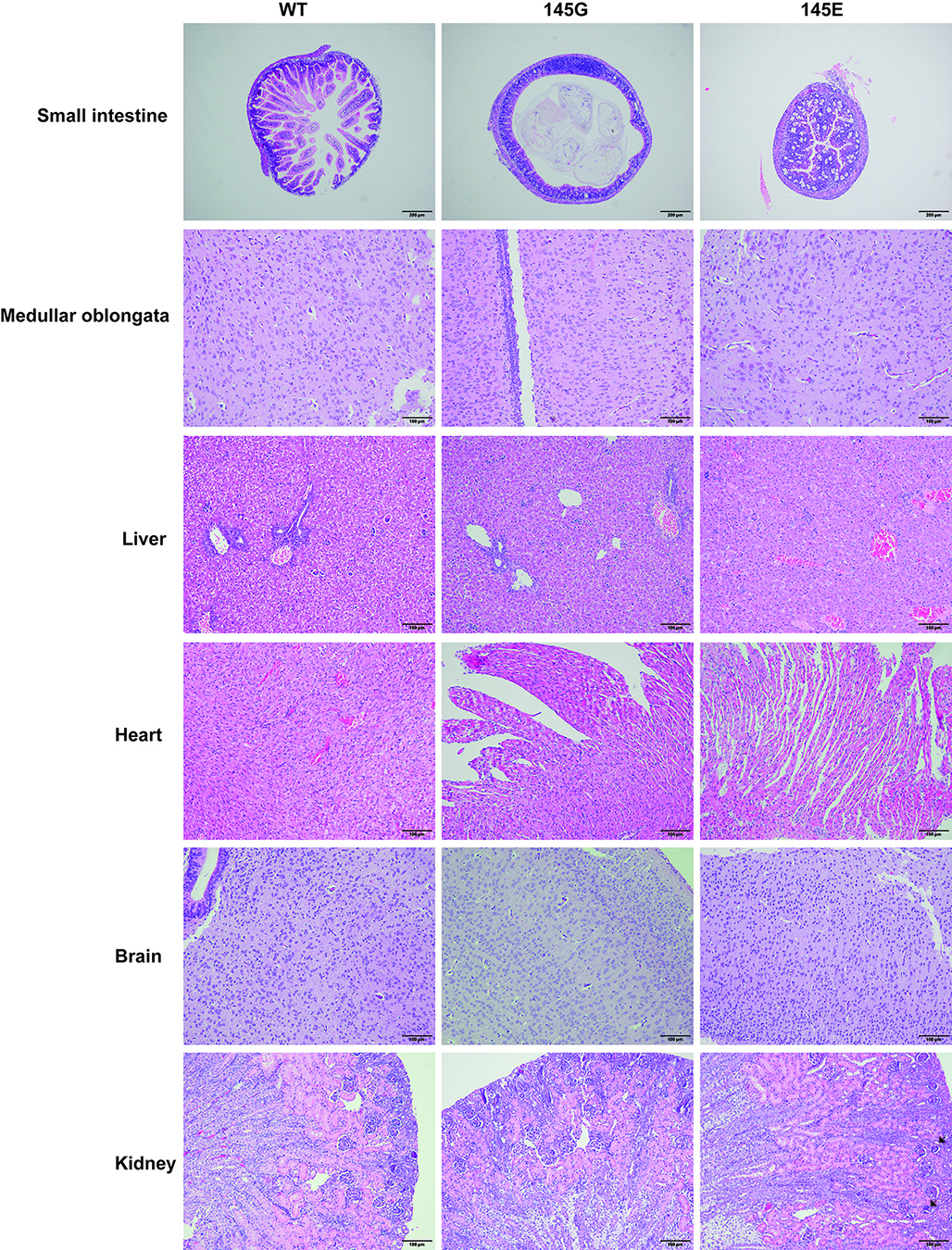

Supplement: Supplemental Material [file TEMI_A_1729665_SM5139.zip › Supple Figure 3R.jpg]
